# SF_6_ Negative Ion Formation in Charge Transfer Experiments

**DOI:** 10.3390/molecules29174118

**Published:** 2024-08-30

**Authors:** Sarvesh Kumar, Masamitsu Hoshino, Boutheïna Kerkeni, Gustavo García, Ghofrane Ouerfelli, Muneerah Mogren Al-Mogren, Paulo Limão-Vieira

**Affiliations:** 1 Atomic and Molecular Collisions Laboratory, CEFITEC-Centre of Physics and Technological Research, Department of Physics, Universidade NOVA de Lisboa, 2829-516 Caparica, Portugal; s.kumar@campus.fct.unl.pt; 2 Chemical Sciences Division, Lawrence Berkeley National Laboratory, One Cyclotron Road, Berkeley, CA 94720, USA; 3 Department of Materials and Life Sciences, Sophia University, Tokyo 102-8554, Japan; masami-h@sophia.ac.jp; 4 ISAMM, Université de la Manouba, La Manouba 2010, Tunisia; boutheina.kerkeni@obspm.fr; 5 Département de Physique, LPMC, Faculté des Sciences de Tunis, Université de Tunis el Manar, Tunis 2092, Tunisia; 6 Instituto de Física Fundamental, Consejo Superior de Investigaciones Científicas (CSIC), Serrano 113-bis, 28006 Madrid, Spain; g.garcia@csic.es; 7 Department of Physics, College of Khurma University, Taif University, P.O. Box 11099, Taif 21944, Saudi Arabia; ghofrane.ouerfelli@fst.utm.tn; 8 Department of Chemistry, College of Sciences, King Saud University, P.O. Box 2455, Ryiadh 11451, Saudi Arabia; mmogren@ksu.edu.sa

**Keywords:** sulphur hexafluoride, charge transfer, anion formation, mass spectrometry, energy loss

## Abstract

In the present work, we report an update and extension of the previous ion-pair formation study of Hubers, M.M.; Los, J. *Chem. Phys.* **1975**, *10*, 235–259, noting new fragment anions from time-of-flight mass spectrometry. The branching ratios obtained from the negative ions formed in K + SF_6_ collisions, in a wide energy range from 10.7 up to 213.1 eV in the centre-of-mass frame, show that the main anion is assigned to SF_5_^−^ and contributing to more than 70% of the total ion yield, followed by the non-dissociated parent anion SF_6_^−^ and F^−^. Other less intense anions amounting to <20% are assigned to SF_3_^−^ and F_2_^−^, while a trace contribution at 32u is tentatively assigned to S^−^ formation, although the rather complex intramolecular energy redistribution within the temporary negative ion is formed during the collision. An energy loss spectrum of potassium cation post-collision is recorded showing features that have been assigned with the help of theoretical calculations. Quantum chemical calculations for the lowest-lying unoccupied molecular orbitals in the presence of a potassium atom are performed to support the experimental findings. Apart from the role of the different resonances participating in the formation of different anions, the role of higher-lying electronic-excited states of Rydberg character are noted.

## 1. Introduction

Sulphur hexafluoride (SF_6_) is an anthropogenic chemical compound that has raised serious concerns across the globe given its role as a potent greenhouse gas and long-term effects when released into the Earth’s atmosphere [1,2]. Such ability to contribute to global warming results from being an efficient infrared absorber within the atmospheric window (≈700–1300 cm^−1^) [3], despite modest contribution to radiative forcing [4]. Its considerably long atmospheric lifetime, up to 3200 years, is results from physical and chemical inertness to atmospheric sink mechanisms such as photodissociation and reaction with radicals [1]. SF_6_ has been widely used as a gaseous dielectric (e.g., in high-voltage lines and particle accelerators), in the semiconductor industry for plasma etching reactors, as well as in a wide range of applied fields where its unique molecular and electronic structure play a relevant role in the underlying electron induced processes yielding a particular reaction channel (see [5] and references therein).

Interaction of low-energy electrons with SF_6_ has attracted the interest of the international scientific community, with several experimental and theoretical studies being reported [5]. SF_6_ is well known to be an efficient electron scavenger because of its large low-energy electron attachment cross-section yielding long-lived SF_6_^−^ ions and higher energy formation of SF_5_^−^ + F [5,6,7,8,9,10,11,12,13,14,15,16,17,18,19,20,21]. Dissociative electron attachment to sulphur hexafluoride also yields SF_4_^−^, SF_3_^−^, SF_2_^−^, F_2_^−^, and F^−^ [5,14,19], while the lifetime of the metastable parent anion has been investigated in a few occasions, with values reported from 10 μs up to 10 ms depending on the ro–vibrational internal energy state, yet with no general agreement within the international scientific community [5,9,11,17,19,20,21]. For a comprehensive description on the lifetimes, see ref. [9].

Electron-induced processes with SF_6_ also include electron scattering absolute elastic differential and total elastic cross-sections (including momentum transfer cross-sections) from experiments [22,23,24,25,26] and theoretical calculations [27,28,29] (and references therein), whereas cross-sections at low energies for vibrationally elastic and inelastic scattering have been calculated using a multichannel effective range theory (ERT) with complex boundary conditions [26]. Formation of SF_6_ negative ions has been investigated in state-selected Rydberg electron transfer experiments [30,31,32,33], while Hubers and Los reported the dependence of ion-pair formation in collisions of fast alkali atoms (K, Na, and Li) from a threshold up to 35 eV [34].

In this contribution, we report the experimental results on SF_6_ anion formation and its fragmentation pattern upon electron transfer in a wide collision energy range together with quantum chemical calculations. Moreover, this is an update and extension of a previous ion-pair formation study [34], where new fragment anions have been assigned and the collision dynamics thoroughly discussed from the ionic yields and the novel energy loss spectrum. In Section 2, we present the results, whereas Section 3 deals with the discussion of the experimental data and the theoretical calculation results needed to support the experimental findings. Section 4 gives a brief description of the experimental apparatus and the computational details. Finally, some conclusions that can be drawn from this study are given in Section 5.

## 2. Results

**Figure 1:**
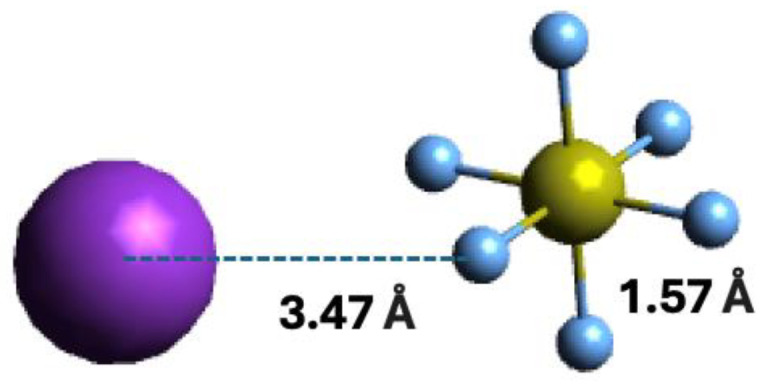
Molecular optimised structure of sulphur hexafluoride, and orientation of the K + SF_6_ collisional system. Colours are purple for K, light blue for F, and yellow for S. Bond lengths are in Å.

Within the scope of negative ion formation in neutral potassium (K)–neutral sulphur hexafluoride (SF_6_) molecule collisions, we make use of theoretical calculations on the lowest unoccupied molecular orbitals (LUMOs) to obtain information about the most accessible electronic states. Thus, SF_6_ electronic structure in the presence of a K atom has been obtained with the shape and energy of the different molecular orbitals calculated up to 21 eV (Section 4). For detailed information on the calculated occupied and virtual Mos; see Table S1. The optimised geometry of the bare molecule and in the presence of the K atom were obtained at the M06-2X/6-311++g(3df,3pd) level of theory, with a K and F distance of 3.47 Å, as depicted in Figure 1.

**Figure 2:**
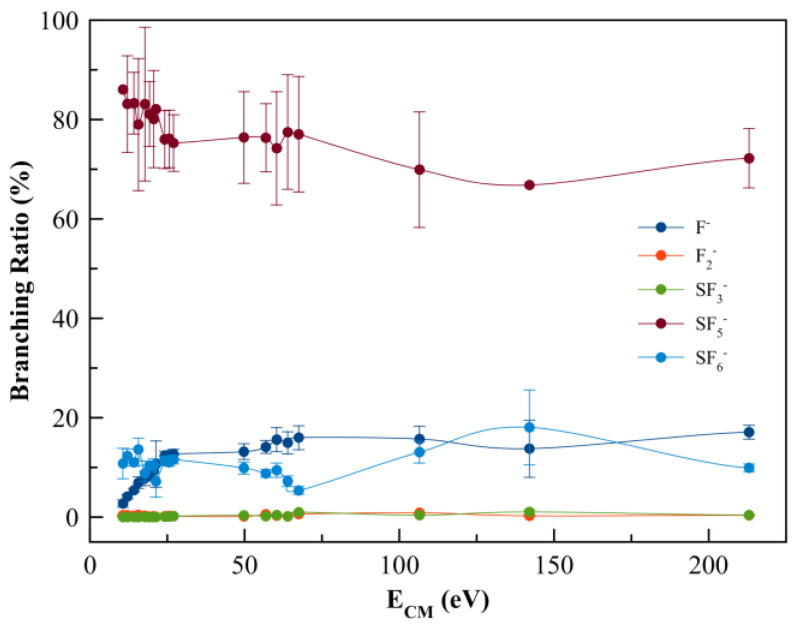
SF_6_ branching ratios (anion yield/total anion yield) from the anions formed as a function of the collision energy in the centre-of-mass frame (CM). Error bars are related to the experimental uncertainty associated with each ion yield. The solid lines are just to guide the eye.

Negative ions formed in electron transfer experiments from K–SF_6_ collisions were mass analysed by TOF mass spectrometry. The wide collision energy range probed in the present study (15–300 eV in the lab frame, 10.7–213.1 eV in the centre-of-mass frame) shows that the main anion is assigned to SF_5_^−^ and contributing to more than 70% of the total ion yield, followed by the non-dissociated parent anions SF_6_^−^ and F^−^ (Figure 2). Above 25 eV, strong competition is noted between the SF_6_^−^ and F^−^ yields, which is reminiscent of the rather high electron affinity of the fluorine atom, while in the case of SF_6_ such is related to the anion’s molecular structure ability to redistribute excess energy through the available internal degrees of freedom. Other minor fragments accounting for less than 1% are due to SF_3_^−^ and F_2_^−^ formation across the entire collision energy range investigated.

**Figure 3:**
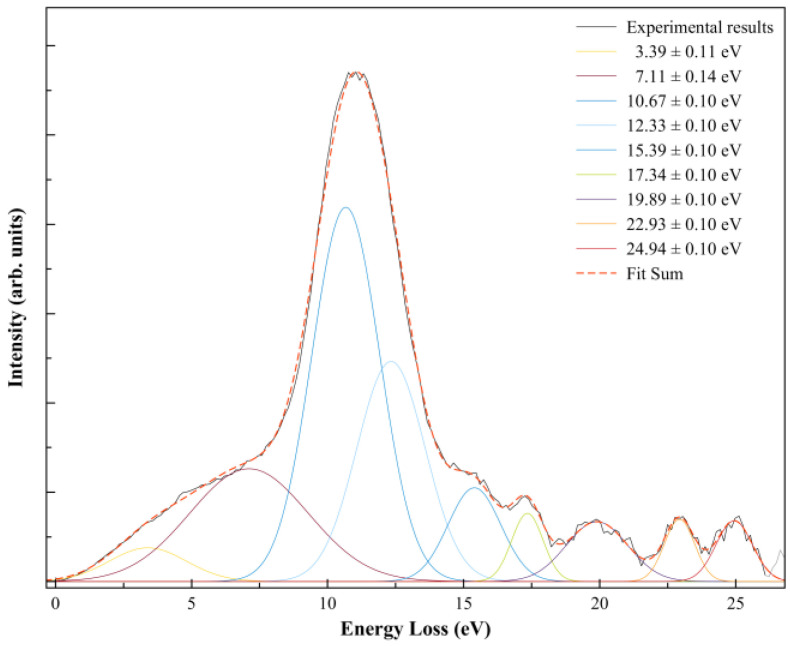
K^+^ energy loss spectrum from K + SF_6_ collisions at ≈146 eV in the centre-of-mass frame (205 eV in the lab frame) in the forward scattering direction (*θ* ≈ 0°). The different decomposed features result from Gaussian fittings with their related uncertainties.

In order to further our knowledge on the nature of the different accessible anionic states, after electron transfer the K^+^ ion formed was energy loss analysed in the forward scattering direction (*θ* ≈ 0°) at a collision energy of ≈146 eV in the centre-of-mass frame, 205 eV in the laboratory frame (Figure 3).

Table 1 lists the vertical electron affinities (VEAs) and assignments of the different features from the Gaussian fitting to the post-collision potassium cation (K^+^) energy loss spectrum. Briefly, in such an ion-pair formation collision process, there is an energy loss (ΔE) feature as follows:

ΔE = IE(K) − EA(I_max_) (1)

with IE(K) being the potassium atom ionisation energy (4.34 eV [35]) and EA(I_max_) the vertical electron affinity of a given state [36,37].

A close inspection of Figure 3 shows a main feature peaking at 10.67 ± 0.10 eV (I_max_) that corresponds to a vertical electron affinity of (−6.33 ± 0.10) eV, which is related to the broad dissociative electron attachment resonance at ~5.4 eV yielding F^−^ [19]. An energy difference of ~0.9 eV is acceptable within the energy resolution of the K^+^ energy loss data and fitting uncertainties.

## 3. Discussion

Following a methodology previously established when dealing with negative ion formation in charge transfer experiments [39,40,41,42,43,44,45,46,47,48,49,50,51,52,53,54,55], the information obtained from electron attachment studies is relevant to assess the role of the main resonances involved, either shape- and/or core-excited. In the electron transfer mechanism:

K + ABC → [K^+^ ABC^−#^] (2)

K is the potassium atom, ABC a polyatomic molecule, and ABC^−#^ a temporary negative ion (TNI) formed with excess internal energy that may yield a stable parent anion or different fragmentation channels. The role of the collision complex formed, [K^+^ ABC^−#^], due to the strong coulomb interaction while the potassium cation is in the vicinity of the TNI, may dictate a different fragmentation pattern (and even anionic yields) from dissociative electron attachment processes.

The collision dynamics are certainly different from the electron attachment (and dissociative electron attachment, DEA) process and TNI autodetachment can be delayed long enough to allow intramolecular energy redistribution within the TNI’s different degrees of freedom. Such a stabilisation process can yield either a non-dissociated parent anion (see, e.g., [50]) or fragment anions from effective bond excision within the TNI (via direct or statistical dissociation). In atom–molecule collisions, electron transfer occurs in the vicinity of the crossing between the ionic (K^+^ + ABC^−^) and the covalent (K + ABC) configurations of the colliding partners. The endoergicity of the process, ΔE, is given by Equation (1), i.e., the ionisation energy of the electron donor and the electron affinity of the target molecule. For a thorough description on ion-pair formation collisional processes, see [36,37] and references therein.

The negative ions formed in K + SF_6_ collisions across the energy range 10.7–213.1 eV in the centre-of-mass frame were assigned to SF_6_^−^, SF_5_^−^, SF_3_^−^, F_2_^−^, and F^−^ (see Figure 2), with SF_5_^−^ as the major contribution. It is worth noting that a contribution barely discernible at 32 u, that can be assigned to O_2_^−^ formation from a leak in the sample system, is also tentatively assigned to S^−^ formation and was not included in the branching ratios of Figure 2, but will be discussed below. We are aware of a previous ion-pair formation work where only SF_6_^−^, SF_5_^−^, F_2_^−^, and F^−^ ions are reported [34], however with no information about K^+^ post-collision energy loss. From the present experimental data and the theoretical calculations, the next sections provide a discussion on the different anions formed and the role of the molecular orbitals participating in the K + SF_6_ electron transfer process.

### 3.1. SF_6_^−^ Formation

The non-dissociated parent anion, together with F^−^, contributes as the second most abundant ion yield across the entire collision energy range investigated (see Figure 2). The reaction that involves parent anion formation is given by Equation (3):

K + SF_6_ → [K^+^ SF_6_^−#^] → K^+^ + SF_6_^−^ (3)

with SF_6_^−#^ a temporary negative ion (TNI) with excess of internal energy. Although SF_6_ is an extremely efficient electron scavenger, and a highly symmetric molecule, its yield does not surpass ~15% of the total anion yield. This is an interesting result when comparing such yield with other polyatomic molecules, e.g., hexachlorobenzene [51] and nitroimidazoles [52,56], where the non-dissociated parent anion is the major ion signal. The branching ratios in Figure 2 show that in the low-energy collision region (<20 eV), the ion signal is mostly due to SF_5_^−^ (>80%), F^−^ (<9%), and a minor contribution of F_2_^−^ (<0.4%). This behaviour is reminiscent of SF_6_^−^ strongly competing with either autodetachment or bond breaking processes. Notwithstanding, and yet not reported here, SF_6_^−^ formation is a dominant anion for collision energies below 10.7 eV, with a threshold at (3.86 ± 0.10) eV [34].

**Figure 4:**
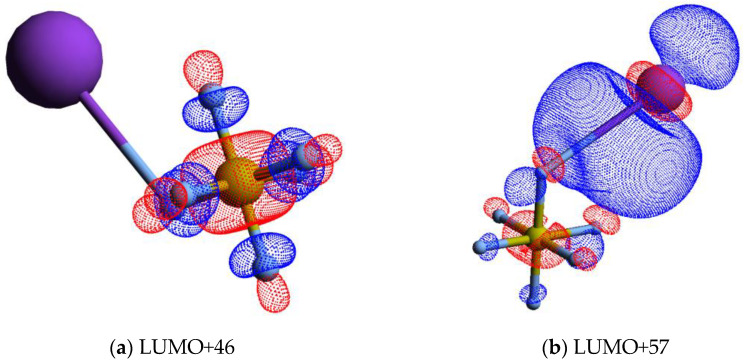

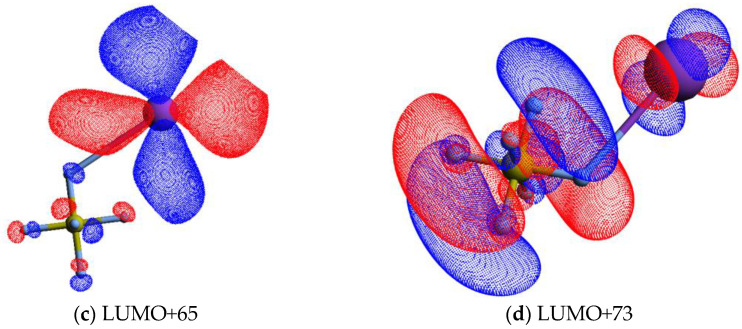
Calculated lowest unoccupied molecular orbitals for K + SF_6_ (K: purple, S: yellow, F: light grey): (**a**) LUMO+46; (**b**) LUMO+57; (**c**) LUMO+65; (**d**) LUMO+73. The K atom and one of the F atoms are connected by a straight line, meaning the spatial mutual position only.

The calculated lowest unoccupied molecular orbitals (LUMOs) in the presence of a potassium atom show a symmetric delocalized spin density over the sulphur atom, with LUMO+46 exhibiting a relevant S–F σ* antibonding character. Given the high symmetry of SF_6_ molecules, strong competition among all six equivalent fluorine atoms for the extra charge are noted (see Figure 4 and also Figure S1), thus yielding either SF_6_^−^ or F^−^.

As the collision energy is increased, more energy may be deposited in the SF_6_ molecule, leading to further fragmentation at the expense of lower SF_6_^−^ yield. However, the non-dissociated parent anion becomes somehow constantly insensitive to the entire collision energy range, albeit with modest changes to within ±5% (Figure 2). This ability of sulphur hexafluoride parent anion yield is related to its electronic structure with a delocalized spin density over all molecules, which is much more discernible from the high-energy LUMO+97 (see Figure S1). Another interesting aspect of the SF_6_ branching ratio, together with the other fragment anions above 25 eV, pertains to the rather insensitive tendency of the yields with the increasing collision energy. Such behaviour can be related to collision dynamics with rather fast collision times (<30 fs) above 75 eV, not allowing sufficient time for K^+^ post-collision to interact with the TNI via a relevant coulomb interaction and thus allowing efficient internal energy redistribution. Vibronic coupling may not play a relevant role regarding the fragmentation yields (see discussion below) and at such high energies the molecular target can be considered as “rigid”. The TNI is mostly formed via the fast and direct vertical access above the neutral ground-state (within the Franck–Condon region) yielding the relevant $\sigma_{SF}^{*}$ antibonding character of the upper ionic states. This agrees with the electron spin densities in Figure 4 and Figure S1. At such higher energies, collision induced fragmentation is dictated by the electron affinity of the different radicals, with particular relevance to SF_5_ and F (see Table 2).

The K^+^ energy loss spectrum at ≈146 eV collision energy in the centre-of-mass frame in the forward scattering direction (*θ* ≈ 0°) is shown in Figure 3. The weak feature peaking at (3.39 ± 0.11) eV yields a positive electron affinity of (0.95 ± 0.11) eV. The asymptotic limit of SF_5_^−^ + F is 0.2 eV [19] above the ground state of the neutral (SF_6_), meaning that the feature at 0.95 eV does not lead to bond excision, resulting in SF_6_^−^ formation. Such electron affinity (0.95 eV) is in excellent agreement with the adiabatic values reported by Menk et al. [60] and Fenzlaff et al. [19], (0.91 ± 0.07) and (1.05 ± 0.10) eV, respectively, whereas Hubers and Los reported an SF_6_ adiabatic electron affinity of (0.32 ± 0.15) eV (at T = 0 K).

### 3.2. SF_5_^−^ and F^−^ Formation

The TOF mass spectra are dominated by SF_5_^−^ formation across the collision energy range of 10.7 up to 213.1 eV in the centre-of-mass frame (see Figure 2). At first glance, this may not be unexpected given the relative high electron affinity of SF_5_ (see Table 2). The complementary reactions with respect to the negative charge that involve SF_5_^−^ and/or F^−^ formation (Equations (4) and (5)) result from an S–F bond breaking within the TNI as:

K + SF_6_ → [K^+^ SF_6_^−#^] → K^+^ + SF_5_^−^ + F (4)

K + SF_6_ → [K^+^ SF_6_^−#^] → K^+^ + SF_5_ + F^−^ (5)

where (SF_5_–F) is a direct bond cleavage and the extra charge sitting either on the SF_5_^•^ or F^•^ radicals.

It is interesting to note that the electron affinities of such radicals are rather identical, i.e., *EA*(F) = (3.401191 ± 0.000026) eV and *EA*(SF_5_) = (3.850 ± 0.020) eV [35], but the SF_5_^−^ yield is over four times higher than the F^−^ signal. This difference cannot be solely explained in terms of electron affinities but rather based on the electronic structure of the TNI formed upon electron transfer. A close inspection of the lowest unoccupied molecular orbitals, where the extra electron can attach, shows LUMO+46 with a strong S–F σ* antibonding character (Figure 4). Rosa et al. [61], with dissociative attachment studies, have demonstrated that SF_6_ excited to the degenerate stretching $\nu_{3}\left(t_{1u}\right)=948\;{cm}^{-1}$ mode, and its molecular structure “leaving out the apex fluorine atom” exhibits a resemblance with that of SF_5_^−^. The SF_5_^−^ branching ratio in Figure 2 shows a rather constant behaviour for 25 < E_CM_ < 70 eV. Note that within the TNI, relevant vibronic coupling may occur in this energy region because the collision time varies from ~33 to ~55 fs, with the upper limit being the same period of the degenerate stretching S–F, $\nu_{3}\left(e\right)=602.5/596\;{cm}^{-1}$ mode in SF_5_^−^ [35]. Moreover, we cannot discard the possibility of stretching S–F, $\nu_{1}\left(a_{1}\right)=795.8/795.5\;{cm}^{-1}$ mode also contributing to such anion formation [6]. Therefore, mode selectivity of $\nu_{3}\left(t_{1u}\right)$ (and/or $\nu_{1}\left(a_{1}\right)$) may be responsible for the relevant SF_5_^−^ yield. Below 25 eV, and at the expense of F^−^, we observe an enhancement in SF_5_^−^ formation (Figure 2).

At higher collision energies, i.e., above 75 eV, one notes a moderate decrease in the SF_5_^−^ ion signal (Figure 2), reminiscent of vibronic coupling being no longer a relevant mechanism in the dissociation dynamics. The molecular target at those energies can be considered rigid, meaning that the excess energy within the TNI will no longer be channelled into the internal degrees of freedom but rather into the main antibonding character MOs with the extra electron sitting favourably on the radical with the higher electron affinity. It is worthy of note that as the collision energy is further increased, so is the relevance of core-excited resonances that may relax into a dissociative state by internal conversion.

The thresholds for reactions in Equations (4) and (5) can be obtained from the SF_5_–F bond dissociation energy [58] and the electron affinities of SF_5_ and F [35] after adding the potassium ionisation energy (4.34 eV [35]), i.e., *D*(SF_5_–F) = (4.00 ± 0.16) eV, *EA*(SF_5_) = (3.850 ± 0.020) eV, and *EA*(F) = (3.401191 ± 0.000026) eV, yielding 4.490 and 4.939 eV. These values are obtained assuming no excess of internal energy (E*) in the process. Hubers and Los [34] report SF_5_^−^ and F^−^ thresholds at (4.85 ± 0.10) and (7.30 ± 0.10) eV. However, the TOF translational excess energy release from the dissociative electron attachment study of Fenzlaff et al. [19] shows F^−^ being formed with thermal or quasi-thermal energies with a mean value < 0.15 eV. If we take this value, the expected thresholds’ upper limits are now obtained at 4.640 (for SF_5_^−^) and 5.089 eV (for F^−^). The energy loss feature peaking at (10.67 ± 0.10) eV (Figure 3) yields a vertical electron affinity of (–6.33 ± 0.10) eV (Table 1). Such an electronic state is calculated at 6.30 eV and can result from access to LUMO+57 with a relevant $\sigma_{SF}^{*}$ antibonding character. Note that DEA experiments report a relevant F^−^ signal with a main resonance peaking at ~5.4 eV (Table 1), whereas SF_5_^−^ only exhibits a single resonance at ~0.5 eV [5]. The energy loss feature with an estimated threshold of ~5.7 eV, i.e., a ~0.6 eV energy difference due to the K^+^ beam energy resolution of the experimental setup (Section 4), can solely be due to an F^−^ signal.

### 3.3. SF_3_^−^, F_2_^−^, and S^−^ Formation

The branching ratios in Figure 2 show that SF_3_^−^ and F_2_^−^ account together for ≲1.2% of the total anion yield. The possible reaction mechanisms related to such an anion’s formation are given by the following:

K + SF_6_ → [K^+^ SF_6_^−#^] → K^+^ + SF_3_^−^ + 3F (6a)

K + SF_6_ → [K^+^ SF_6_^−#^] → K^+^ + SF_3_^−^ + F_2_ + F (6b)

K + SF_6_ → [K^+^ SF_6_^−#^] → K^+^ + F_2_^−^ + SF_4_ (7)

Note that in Equations (6a) and (6b), the loss of three fluorine atoms may proceed through reactions yielding F + F + F and/or F_2_ + F, with calculated thresholds of 11.250 and 9.623 eV. In the energy loss data in Figure 3, the feature peaking at (15.39 ± 0.10) eV, with an estimated appearance at (11.56 ± 0.10) eV, may be related to reaction (6a) within the experimental uncertainties. However, we do not discard the possibility that the energy loss feature peaking at (12.33 ± 0.10) eV can also be responsible for reaction (6b). The information obtained from the DEA data of Fenzlaff et al. [19] shows SF_3_^−^ with a wide resonance, a maximum at ~11 eV, and a threshold of ~9 eV, much more consistent with SF_3_^−^ + F_2_ + F formation. Whether in electron transfer yielding a negative ion favours reaction (6a) against DEA to SF_6_, leading to SF_3_^−^ + F_2_ + F, still remains to be proven within the framework of the collision dynamics in K + SF_6_.

We look at reaction (7), where F_2_^−^ formation from SF_6_^−#^ requires the excision of two S–F bonds and fluorine has to be formed. From F_2_ electron affinity (3.005 ± 0.071) eV [35] and the bond dissociation energies from Table 2, *D*(SF_5_–F) = (4.00 ± 0.16) eV, *D*(SF_4_–F) = (2.51 ± 0.13) eV, and *D*(F–F) = (1.627 ± 0.100) eV, and after adding the potassium ionisation energy of 4.34 eV, the appearance energy of reaction (7) is given by $\varepsilon_{th}\left(F_{2}\right)$ = *D*(SF_5_–F) + *D*(SF_4_–F) − *D*(F–F) − *EA*(F_2_) + IE (K) = (6.218 ± 0.150) eV. The energy loss data in Figure 3 at (10.67 ± 0.10) eV shows an estimated threshold at ~(7.34 ± 0.10) eV, in reasonable agreement within the K^+^ beam energy resolution.

Finally, we detain ourselves on the contribution within the TOF mass spectra of the fragment anion 32 u. Such an anion can either originate from electron transfer to O_2_ present from a small leak in the sample system or can be assigned to S^−^ formation. In order to assess the origin of such an anion, we have comprehensively compared the background yields of O_2_^−^ and O^−^ with the total cross-section for negative ion formation in K + O_2_ collisions from the benchmarking work of Kleyn et al. [62]. At 20 eV in the centre-of-mass frame, the cross-section ratio O^−^/O_2_^−^ is ~0.67 [62]. Although our TOF mass spectrum at such collision energy shows no traces of 32 u anion formation, while at 22 eV a barely detectable signal is discernible from the background. The expected ratio of O^−^/O_2_^−^ at 22 eV is ~0.63 [62], which is not noted in the present experiments, thus suggesting S^−^ formation. The reaction yielding S^−^ requires considerable intramolecular processes, with six S–F bonds to be broken and the extra charge sitting on the sulphur atom. Such a process seems unlikely to happen, at least from the DEA data available in the literature [5]. However, we have observed on several occasions that within the framework of potassium–molecule collisions either the fragmentation pattern or different anions being formed differ from electron attachment/dissociative electron attachment experiments [44]. The differences have been put forward on the role of the collision complex (Equation (2)), with the K^+^ ion being a relevant partner allowing stabilization of the TNI while in its vicinity before leaving the collision region. Thus, under particular energy constraints, the role of the electronic, and the structure of the molecular target, different fragmentation channels can be accessed. The calculated threshold of formation with a lower limit at ~ 18.1 eV is obtained from the bond dissociation energies and the sulphur electron affinity (Table 2), assuming no excess of energy (E*). Within such dissociative electron attachment experiments, we observe in the ion yields above 14 eV a continuous increase in the background signal, which is reminiscent of ion-pair formation [19]. If such anions were to be formed, the expected low yield would probably not become discernible from such a continuum. In the presence of a potassium atom, the threshold of S^−^ reaction is now expected at ~22.4 eV, which seems to be in good agreement with the TOF mass spectrometry data at that collision energy. An energy loss feature with a maximum at (22.93 ± 0.10) eV (Table 1) shows a threshold at ~21.1 eV (Figure 3). The calculated vertical energy (17.60 eV) is in reasonable agreement with the experimental value of (–18.59 ± 0.10) eV (Table 1) to within the energy loss resolution. From the theoretical calculation, this was assigned to the contribution of LUMO+101 (see Figure S1), where a strong antibonding character is visible along the S–F bonds, becoming more relevant at higher energies, as is the case for LUMO+102. Electron promotion to these MOs may yield S^−^.

### 3.4. Energy Loss Data

The energy loss spectrum in Figure 3 was smoothed and fitted with Gaussian functions to decompose the energy loss spectrum, with vertical electron affinities and assignment of the main MOs in Table 1. Different fittings up to (15.39 ± 0.10) eV were obtained from the energy position of the resonances from DEA experiments [5] while accounting for the width of the Gaussian fittings with the related energy resolution of the charge transfer experiment. Assignments above 15 eV were performed based on information from quantum chemical calculations, with the nature of the most relevant MOs depicted in Figure 4 and Figure S1. Of relevance are the electronic-excited states of SF_6_ with vertical electron affinities above 11 eV that can be associated with features converging to different ionisation limits, 15.890 eV(^2^*T*_1g_), 16.938 eV(^2^*T*_1u_), 17.360 eV(^2^*T*_2u_), 18.434 eV(^2^*E*_g_), and 22.7 eV(^2^*T*_1u_) [63,64], and so promotion of an electron to higher orbitals of increasing Rydberg character are noted. These are the cases of LUMO+73 in Figure 4, and all MOs in Figure S1. These higher-lying energy MOs show delocalized electron spin densities evocative of such character, and from different ionisation energies, the MOs can be tentatively assigned to *n*s, *n*p, and *n*d. At these energies, the number of electronic states is considerably large, making an unambiguous assignment difficult, so these can also be listed as (*n* + 1) or (*n* + 2).

A close inspection of the lowest unoccupied molecular orbitals, where the extra electron can attach, shows LUMO+46 with a strong S–F σ* antibonding character (Figure 4). The calculated vertical energy is 3.81 eV, a value quite higher than the related resonance positions of F^−^ and F_2_^−^ from DEA experiments (Table 1) [5]. Notwithstanding, the electron spin densities are in agreement with the favourable bond excision yielding F^−^, while F_2_^−^ formation certainly may require different dynamics within the TNI formed after electron transfer. We have recently observed in the case of C_6_Cl_6_ [65] a strong vibronic coupling in the TNI involving the C–Cl bending motion, where the chlorine atoms are brought together within the ring framework due to excess energy dissipation producing Cl_2_^−^. Whether this mechanism is also operative in SF_6_ stands to be proven but is certainly beyond the scope of the present contribution.

Recently, we have investigated different polyatomic molecular targets, viz., CH_3_OH [55], H_2_O/D_2_O [53], and C_2_H_5_OH [41], where we stressed that the output of the calculations performed providing information on electronically excited states are related to single occupied MO being replaced by a non-occupied (virtual) MO only. Therefore, we are not able to account for the role of doubly excited states, yet these can certainly be considered, in particular given the energy loss features observed at higher energies. This will be crucial to evaluating the relevant competition between super-excited states and bond excision into neutral fragments (and even dipolar dissociation), which has been noted for H_2_O/D_2_O energy loss features above 17 eV [53].

## 4. Materials and Methods

Ion-pair formations in collisions between neutral potassium atoms and neutral SF_6_ molecules were been obtained in a crossed molecular beam apparatus described elsewhere [50,53,54,66]. Briefly, the Lisbon apparatus (see Figure S2) comprises two interconnected and differentially pump vacuum chambers with base pressures in the potassium chamber of 4 × 10^−5^ Pa and in the collision chamber of 5 × 10^−5^ Pa. After SF_6_ admission in the collision chamber, the working pressure was 1 × 10^−3^ Pa for both time-of-flight (TOF) mass spectrometry and K^+^ energy loss measurements. A beam of hyperthermal neutral potassium atoms is produced by resonant charge exchange between K^+^ ions and thermal potassium atoms in a charge exchange oven (CEO). The ion beam is delivered from a commercial ion source (HeatWave, USA) that is accelerated to a set kinetic energy towards the entrance of the CEO, while thermal potassium atoms are obtained by heating solid potassium at 393 K. From such an interaction, the resultant beam comprises hyperthermal ions that did not charge exchange and are removed from the hyperthermal neutral beam by a pair of deflecting plates placed outside the CEO, before traveling into the collision region. From the resonant charge-exchange process and the CEO slit apertures, the hyperthermal neutral beam is mainly composed of K atoms in the ground-state with its outermost electron as 4s. This has been previously checked in other energy loss data from potassium collisions with pyrimidine [46], halothane [67], tetrachloromethane [66], and more recently with hexachlorobenzene [51,68], nimorazole [52], water [53], methanol [55], and ethanol [41]. Of relevance, the experimental thresholds of formation are in assertion of K in a 4s state rather than in a 4p state, which otherwise would result in values at lower energies than those reported here (see Section 3). The hyperthermal neutral potassium beam intensity is monitored at the entrance of the collision chamber by a surface ionisation Langmuir–Taylor detector. The beam is made to cross at right angles with an effusive SF_6_ beam, which is admitted to vacuum through a 1 mm diameter capillary from an external sample holder.

The negative ions formed in the collision region were extracted by a pulsed electrostatic field (380 Vcm^−1^), and mass analysed in a dual-stage linear TOF with a mass resolution m/Δm ≈ 125. The potassium beam energy resolution for TOF mass spectra collection in the collision energy range investigated was ∼0.6 eV. The TOF mass calibration was performed from well-known fragmentation patterns from collisions of potassium atoms with CH_3_NO_2_ and/or CCl_4_ molecules [50,66]. Note that comprehensive background spectra (without the sample) were obtained and subtracted from the sample measurements. Branching ratios (BRs) for the fragment anions were obtained and result from the fragment anion yield divided by the total anion yield at a given collision energy.

Potassium cations formed in post-collision experiments were energy loss analysed in the forward scattering direction (*θ* ≈ 0°), while experiments were not performed in coincidence with TOF mass spectrometry. The analyser was operated in constant transmission mode, hence keeping the resolution constant throughout the entirety of the scans. The estimated energy resolution during the experiments was ~1.2 ± 0.2 eV. The energy loss scale was calibrated using the K^+^ beam profile from the potassium ion source serving as the “elastic peak”. SF_6_ was supplied by Alphagaz with a stated purity ≥ 99.9% and was used as delivered.

Vertical excitation energies of the lowest unoccupied molecular orbitals (LUMOs) of SF_6_ in the presence of a K atom were calculated to assess the nature of the different electronic states. The geometry of a sulphur hexafluoride + potassium system was optimised at the M06-2X/6-311++g(3df,3pd) level of theory and the equilibrium mutual distance between the potassium K and fluorine F atoms is 3.47 Å. The coordinates of the resulting optimised system are depicted in Figure 1. Quantum chemical calculations were performed with the Gaussian 16 program package [69] and carried out in Cartesian coordinates, with no symmetries. All electrons were considered for sulphur, fluorine, and potassium atoms with the 6-311++g(3df,3pd) basis set. The natural molecular orbitals for K−SF_6_ were calculated by the same model chemistry.

## 5. Conclusions

We have performed electron transfer experiments in potassium–sulphur hexafluoride collisions in the centre-of-mass energy range from 10.7 to 213.1 eV (15–300 eV in the laboratory frame). The different time-of-flight mass spectra recorded allowed extending and updating the previous ion-pair formation study of Hubers and Los [34] with new fragment anions assigned, namely, SF_6_^−^, SF_5_^−^, SF_3_^−^, F_2_^−^, and F^−^. Moreover, from the mass spectrometry data, a tentative assignment of anion 32u is suggested to be S^−^, although there would be a rather complex intramolecular mechanism within the TNI yielding such an ion. The main anion across the entire collision energy range is assigned to SF_5_^−^, with >70% of the total anion yield, in strong contrast to electron attachment experiments yielding the intact parent anion, SF_6_^−^. In the low-energy collision region, the branching ratios show a relevant dependence where the excess internal energy deposited in the TNI leads to different fragmentation channels. The anions formed have been discussed with the help of quantum chemical calculations from the shape of the electron spin densities in the different MOs accessed during the collision process. We have made use of the K^+^ post-collision energy loss data recorded in the forward scattering direction to infer on the electronic state spectroscopy of SF_6_. The information available in the literature from electron attachment experiments and theoretical data have been critical to identifying the different resonances involved in the charge transfer process. The role of higher-lying excited states with Rydberg character was assessed from the energy loss features above 11 eV. However, due to the lack of further information in the literature, no attempt was made to assign the role of possible doubly excited states, which may be attained at such high collision energies.

## Figures and Tables

**Table 1:** Assignment of different features from Gaussian fittings to K^+^ energy loss spectrum from K + SF_6_ collisions at ≈146 eV in the centre-of-mass frame *. VEA (vertical electron affinity), VE (vertical energy), EA (electron attachment).

K^+^ Energy Loss Feature (eV)	VEA (eV)	Calculated VE (eV)	Assignment	EA resonances (eV) [5]
3.39 ± 0.11	0.95 ± 0.11	–	–	–
7.11 ± 0.14	−2.77 ± 0.14	3.81	LUMO+46	2.6 (2.8; ~2.9 [19])
10.67 ± 0.10	−6.33 ± 0.10	6.30	LUMO+57	~5.4; (5.7 ± 0.1 [38])
12.33 ± 0.10	−7.99 ± 0.10	7.24	LUMO+65	8.8 (8.9)
15.39 ± 0.10	−11.05 ± 0.10	11.01	LUMO+73	11.3 (11.5; 11.6)
17.34 ± 0.10	−13.00 ± 0.10	12.89	LUMO+81	–
19.89 ± 0.10	−15.55 ± 0.10	15.68	LUMO+97	–
22.93 ± 0.10	−18.59 ± 0.10	17.60	LUMO+101	–
24.94 ± 0.10	−20.60 ± 0.10	21.56	LUMO+102	–

* The uncertainties result from the Gaussian fitting procedure.

**Table 2:** Electron affinity and bond dissociation energy (at 0 K) relevant in electron attachment to SF_6_. See text for details.

Compound	Electron Affinity (eV) [35]	Bond Dissociation Energy (eV) [57]
SF_6_	0.910 ± 0.070 ^1^	–
SF_5_	3.850 ± 0.020	–
SF_3_	3.070 ± 0.020	–
F_2_	3.005 ± 0.071	–
F	3.401191 ± 0.000026	–
SF_5_–F	–	4.00 ± 0.16 [58]
SF_4_–F	–	2.51 ± 0.13
SF_3_–F	–	3.47 ± 0.56
SF_2_–F	–	2.64 ± 0.12
SF–F	–	3.98 ± 0.24
S–F	–	3.51 ± 0.07
F–F	–	1.627 ± 0.100 ^2^ [59]

^1^ adiabatic value; ^2^ at 298 K.

## Data Availability

The data that support the findings of this study are available from the corresponding authors upon reasonable request.

## Supplementary Materials

The following supporting information can be downloaded at: https://www.mdpi.com/article/10.3390/molecules29174118/s1. Results from theoretical calculations for the shape of a selection of K + SF_6_ lowest unoccupied molecular orbitals and SF_6_ temporary negative ion (TNI) lowest lying electronic states in the presence of K. Figure S1: Calculated lowest unoccupied molecular orbitals for K + SF6 (K: purple, S: yellow, F: light grey): (a) LUMO+81; (b) LUMO+97; (c) LUMO+101; (d) LUMO+102; Figure S2: Schematic overview of the crossed molecular beam setup for electron transfer experiments; Table S1: Calculated occupied (O) and virtual (V) molecular orbitals of K + SF6 at the M06-2X/6-311++g(3df,3pd) level of theory.
