# Supplementary material for: SF6 Negative Ion Formation in Charge Transfer Experiments
Source: Molecules. 2024 Aug 30;29(17):4118. doi: 10.3390/molecules29174118 (PMC11397648; doi:10.3390/molecules29174118)
Supplement: Supplementary file 1 [file molecules-29-04118-s001.zip › molecules-3155697-supplementary.pdf]

Supplementary Materials

SF_6 negative ion formation in charge transfer experiments

Sarvesh Kumar [1,2], Masamitsu Hoshino [3], Boutheïna Kerkeni [4,5], Gustavo García [6], G. Ouerfelli [7], M Mogren Al-Mogren [8] and Paulo Limão-Vieira [1,*]

[1] Atomic and Molecular Collisions Laboratory, CEFITEC, Department of Physics, Universidade NOVA de Lisboa, 2829-516 Caparica, Portugal; s.kumar@campus.fct.unl.pt (S.K.); plimaovieira@fct.unl.pt (P.L.-V.)
[2] Chemical Sciences Division, Lawrence Berkeley National Laboratory, One Cyclotron Road, Berkeley, 94720, California, USA; skumar2@lbl.gov (S.K.)
[3] Department of Materials and Life Sciences, Sophia University, Tokyo, 102-8554 Japan; masami-h@sophia.ac.jp (M.H.)
[4] ISAMM, Université de la Manouba, La Manouba 2010 Tunisia; boutheina.kerkeni@obspm.fr (B.K.)
[5] Département de Physique, LPMC, Faculté des Sciences de Tunis, Université de Tunis el Manar, Tunis 2092, Tunisia; boutheina.kerkeni@obspm.fr (B.K.)
[6] Instituto de Física Fundamental, Consejo Superior de Investigaciones Científicas (CSIC), Serrano 113-bis, 28006 Madrid, Spain; g.garcia@csic.es (G.G.)
[7] Department of Physics, College of Khurma University, Taif University, P.O. Box 11099, Taif, 21944, Saudi Arabia; ghofrane.ouerfelli@fst.utm.tn (G.O.)
[8] Department of Chemistry, College of Sciences, King Saud University, P.O. Box 2455, Ryiadh, 11451, Saudi Arabia; mmogren@ksu.edu.sa (M.M.)
* Correspondence: plimaovieira@fct.unl.pt (P.L.-V.); Tel.: (+351 21 294 78 59), boutheina.kerkeni@obspm.fr (B.K.)

Figure caption

Figure S1: Calculated lowest unoccupied molecular orbitals for K + SF_6 (K: purple, S: yellow, F: light grey): (**a**) LUMO+81; (**b**) LUMO+97; (**c**) LUMO+101; (**d**) LUMO+102. The K atom and one of the F atoms are connected by a straight line meaning the spatial mutual position only.

Figure S2: Schematic overview of the crossed molecular beam setup for electron transfer experiments.

Table caption

Table S1: Calculated occupied (O) and virtual (V) molecular orbitals of K + SF_6 at the M06-2X/6-311++g(3df,3pd) level of theory.

Figure S1: Calculated lowest unoccupied molecular orbitals for K + SF_6 (K: purple, S: yellow, F: light grey): (**a**) LUMO+81; (**b**) LUMO+97; (**c**) LUMO+101; (**d**) LUMO+102. The K atom and one of the F atoms are connected by a straight line meaning the spatial mutual position only**.**

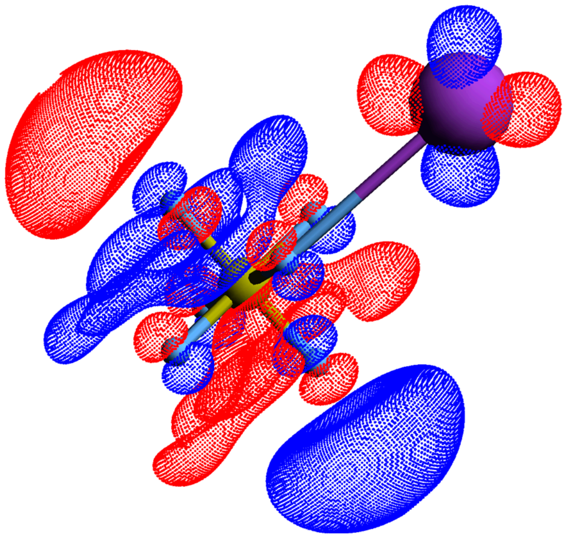

(**a**) LUMO+81

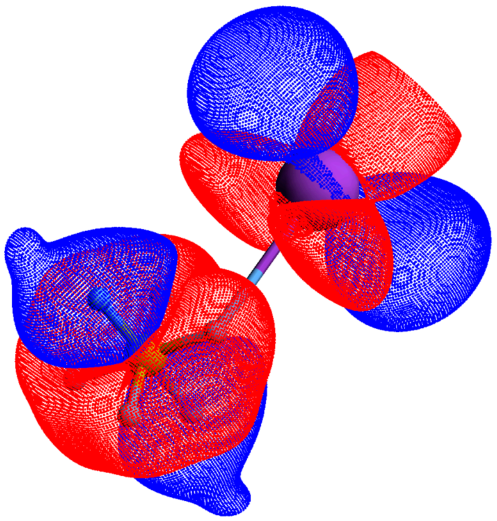

(**b**) LUMO+97

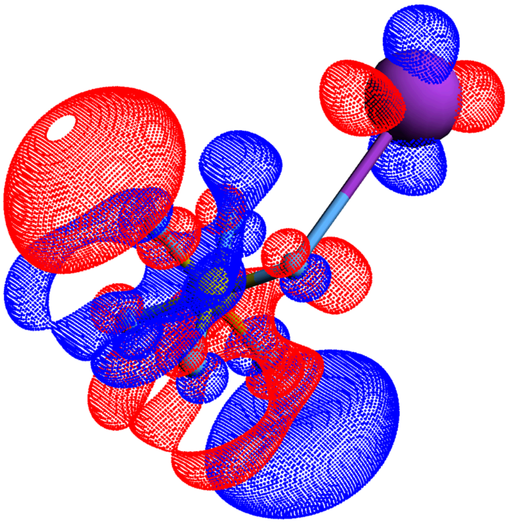

(**c**) LUMO+101

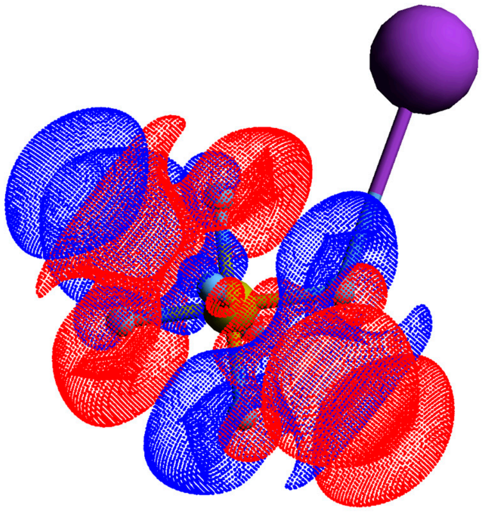

(**d**) LUMO+102

Figure S2: Schematic overview of the crossed molecular beam setup for electron transfer experiments.

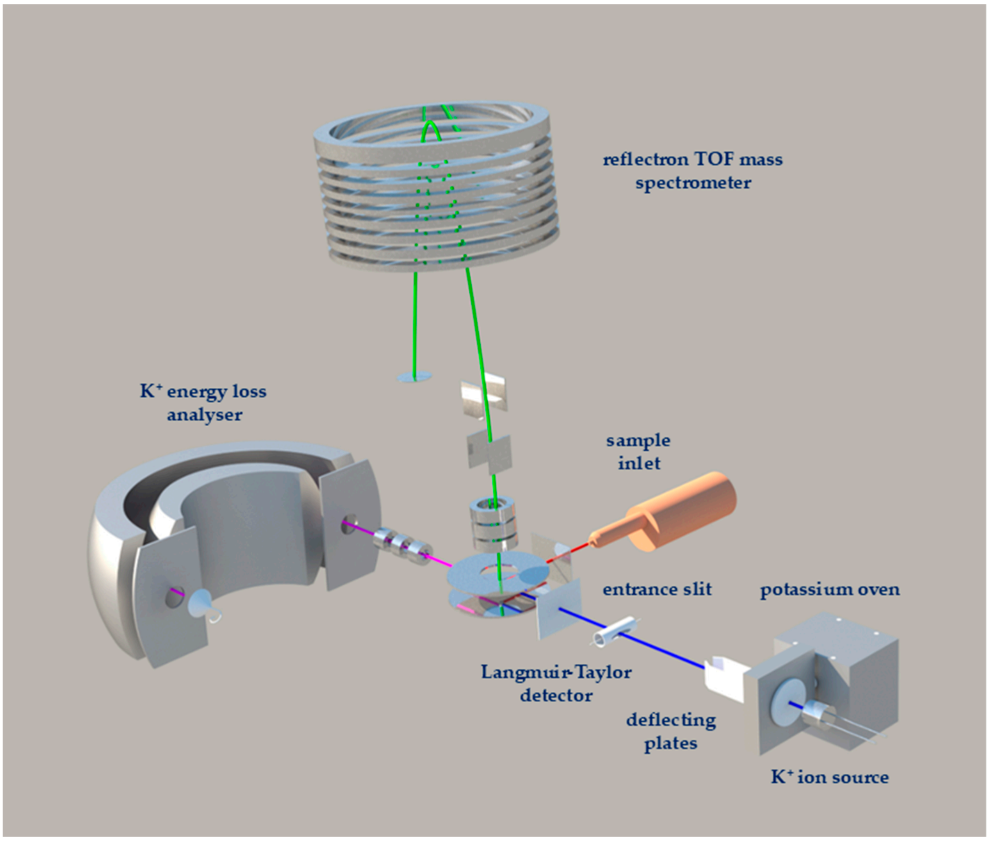

Table S1: Calculated occupied (O) and virtual (V) molecular orbitals of K + SF_6 at the M06-2X/6-311++g(3df,3pd) level of theory.

	Molecular Orbitals	Energy (eV)
1	O	-3561.961839
2	O	-2453.755777
3	O	-684.7461736
4	O	-684.7458198
5	O	-684.715071
6	O	-684.7147172
7	O	-684.6947985
8	O	-684.6943631
9	O	-365.6282897
10	O	-292.638961
11	O	-292.6389066
12	O	-292.6383895
13	O	-230.8351232
14	O	-174.3747063
15	O	-174.3734546
16	O	-174.3716586
17	O	-38.56628245
18	O	-36.53639364
19	O	-35.15000002
20	O	-35.1372923
21	O	-35.13302011
22	O	-33.69571396
23	O	-33.67535983
24	O	-21.20067446
25	O	-18.1688347
26	O	-18.16235838
27	O	-18.16165089
28	O	-17.07439219
29	O	-17.0720248
30	O	-17.04024188
31	O	-14.50296931
32	O	-14.48778535
33	O	-14.47801646
34	O	-12.81950884
35	O	-12.79771251
36	O	-11.82803427
37	O	-11.82003412
38	O	-11.80460525
39	O	-11.73665839
40	O	-11.7321413
41	O	-11.71507975

42	O	-10.58232359
43	O	-10.55086721
44	O	-10.5470304
45	O	0.838300991
46	V	3.805132722
47	V	4.025300159
48	V	4.036837793
49	V	4.119043432
50	V	4.423783901
51	V	4.513091715
52	V	4.543976654
53	V	4.909017585
54	V	5.610663534
55	V	5.643317214
56	V	5.658256273
57	V	6.296064278
58	V	6.70894285
59	V	6.722711818
60	V	6.785189193
61	V	6.914334497
62	V	6.956729858
63	V	7.05297658
64	V	7.139155084
65	V	7.236490262
66	V	8.204046011
67	V	9.166649286
68	V	9.185452364
69	V	9.197398168
70	V	9.701326085
71	V	10.01823005
72	V	10.72197128
73	V	11.0127523
74	V	11.50669363
75	V	11.54544266
76	V	11.57270849
77	V	12.15258342
78	V	12.5389853
79	V	12.5435296
80	V	12.64731388
81	V	12.8879715
82	V	13.11300978
83	V	13.1166289
84	V	13.46278512
85	V	13.59323657
86	V	13.81231555
87	V	14.3757548
88	V	14.43330691

89	V	14.75780286
90	V	14.8261851
91	V	14.86558721
92	V	15.08042121
93	V	15.13797333
94	V	15.27479224
95	V	15.39816873
96	V	15.5479947
97	V	15.68263671
98	V	16.42031055
99	V	17.53230441
100	V	17.5711895
101	V	17.60449626
102	V	21.56440803
103	V	21.58840848
104	V	22.03369583
